# Supraclavicular nerve sparing versus sacrifice during open reduction internal fixation of acute midshaft clavicle fracture

**DOI:** 10.1186/s13018-023-04220-7

**Published:** 2023-09-25

**Authors:** Ruei Hu, Yu-Jung Su, Chi-Sheng Chien

**Affiliations:** 1 Orthopedics Department, Chi-Mei Medical Center, Tainan, Taiwan; 2 Department of Orthopaedics, Kaohsiung Municipal Ta-Tung Hospital, KaoHsiung, Taiwan

**Keywords:** Clavicle fracture, Open reduction internal fixation of clavicle fractures, Supraclavicular nerve sparing, WALANT, Clavicle fracture outcomes

## Abstract

**Background:**

The branches of the supraclavicular nerve are often sacrificed during open reduction and internal fixation (ORIF) for clavicle fracture. No consensus exists on whether the supraclavicular nerve should be routinely identified and protected during ORIF.

**Methods:**

We developed a simple method to make nerve sparing easier; Wide-Awake Local Anesthesia No Tourniquet (WALANT) solution is locally injected prior to the surgical incision being made. This retrospective study enrolled 340 patients and divided them into supraclavicular-nerve-sparing (*n* = 45) and supraclavicular-nerve-sacrifice (*n* = 295) groups. Surgical outcomes—including operative time, estimated blood loss, postoperative pain, union rate, time to union, functional score, paresthesia, complications, implant removal rate, and complication rate—were recorded.

**Results:**

Incisional or anterior chest wall numbness and intraoperative blood loss were significantly less (*p* < 0.001) in the nerve-sparing group. The operative time was similar in the two groups. No significant differences were discovered in QuickDASH score, postoperative pain score, union rate, time to union, implant removal rate, complication rate, or revision rate.

**Conclusions:**

Our study demonstrated that the outcomes of supraclavicular nerve sparing during ORIF with WALANT can reduce postoperative incisional and anterior chest wall numbness and intraoperative blood loss without increasing the operative time or complication rate.

## Introduction

Acute midshaft clavicle fractures are common and can cause significant pain and disability. The incidence of clavicle fractures has increased in recent years and so has the number of surgeries performed for these fractures [1].

The supraclavicular nerve is a superficial sensory nerve originating from the C3 and C4 nerve roots of the superficial cervical plexus, and it innervates the clavicle, anteromedial shoulder, and proximal chest [2]. It usually divides into a medial and a lateral branch, both of which cross the operative field if an incision is made for fixation of a midshaft clavicle fracture [3]. A cadaveric study indicated a predictable pattern of two or three nerves crossing the clavicle in 97% of specimens [2]. Another cadaveric study revealed 7 branch patterns. They pointed out the safe zones were 6.1 mm among both sexes of the SC joint medially, 0.7 mm among females, and 0 mm among males of the AC joint laterally. They concluded that surgical incisions between 29.3 and 51.2% and 60.5 to 79.7% of the clavicle length from the SC joint were the safe zones at the midclavicular shaft among both sexes [4]. These two studies implied that these branches will almost always be encountered during open reduction and internal fixation (ORIF) of clavicle fracture.

Sacrificing these branches can result in sensory deficits in corresponding region and even pain. The incidence of postoperative numbness can be as high as 55–86% [5, 6]. However, preserving these nerves can be challenging because of the vertical relationship between the nerve and the commonly used anterior approach. Moreover, the nerve may obscure fracture reduction, especially in complex fracture patterns.

Recently, a newer technique called Wide-Awake Local Anesthesia No Tourniquet (WALANT) has been increasingly used by hand surgeons [7]. WALANT involves the injection of lidocaine and epinephrine for local anesthesia and vasoconstriction, respectively, before incision and dissection are performed, thereby minimizing bleeding. The less the surgical field bleeding, the easier is the nerve sparing.

Therefore, this study compared the outcomes of supraclavicular nerve sparing with preincisional WALANT local injection with those of supraclavicular nerve sacrifice during ORIF of midshaft clavicle fracture in adults. We hypothesized that the supraclavicular-nerve-sparing group would have less postoperative numbness than and comparable surgical outcomes to the supraclavicular-nerve-sacrifice group.

## Materials and methods

### Patients

This retrospective study was conducted at a single medical center (Chi-Mei Medical center, Tainan, Taiwan), and the study protocol was approved by the institutional review board.

We included all adult patients who underwent ORIF with dynamic compression plate or locking compression plate for midshaft clavicle fracture from May 2020 to September 2022; patients also had to have been followed up for ≥ 6 months. We excluded patients who were under 18 years old; had undergone simultaneous surgery other than clavicular ORIF; had multiple fractures, polytrauma, distal or proximal clavicle fracture, open fracture, chronic fracture (> 4 weeks of injury), or previous clavicular surgery or deformity; and were lost to follow-up.

### Study design

Patient characteristics, including age, sex, body mass index (BMI), side of injury, fracture, fracture classification (AO/OTA), trauma mechanism, time from injury to surgery, and implant type (dynamic or locking compression plate), were recorded.

### Surgical technique

All the surgeries in the nerve-sparing group were conducted by either of the two authors (Dr. Ruei Hu and Dr. Yu-Jung Su). The surgeries in the nerve-sacrifice group were performed by other orthopedic surgeons in our facility.

**Fig.1:**
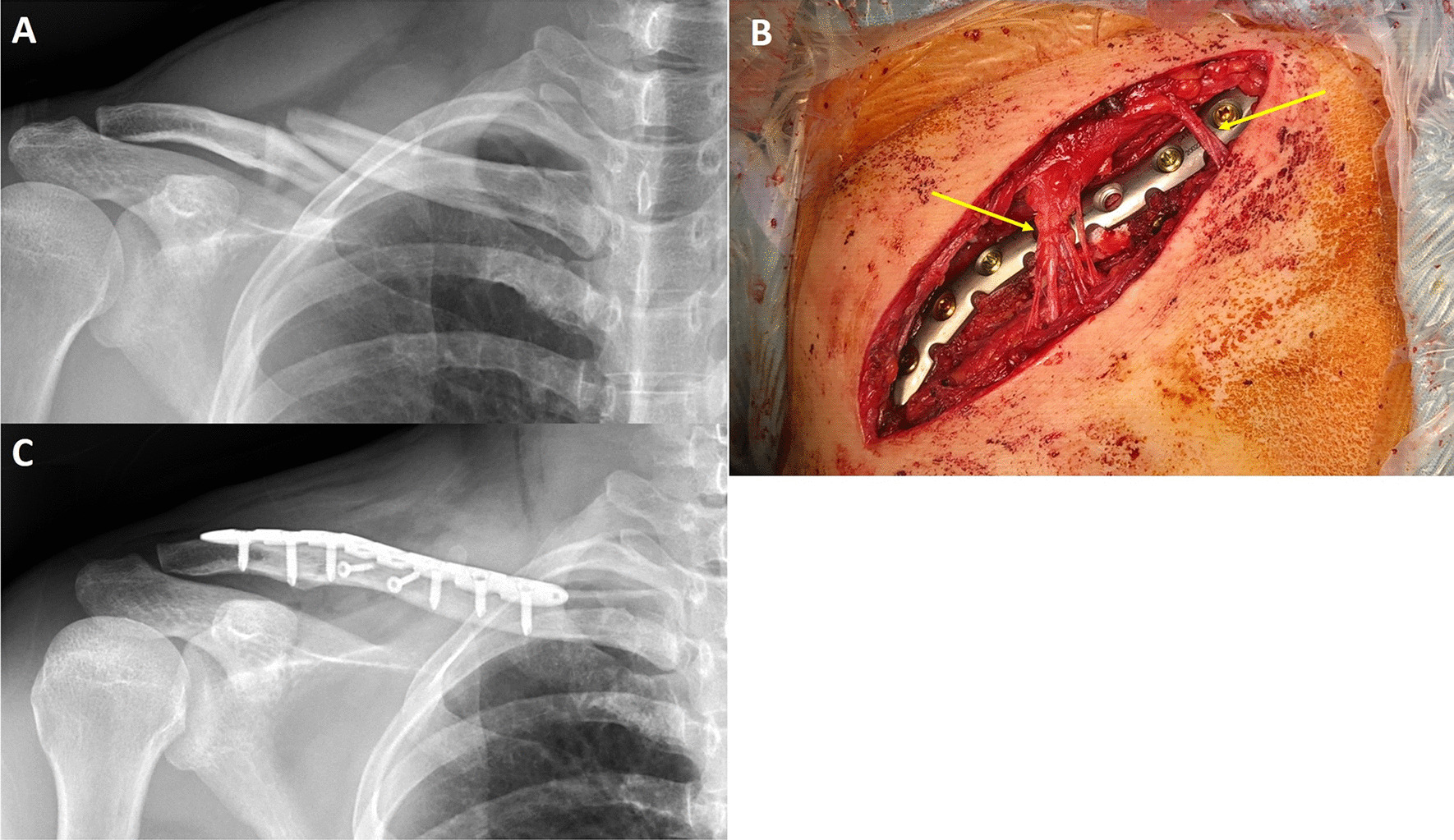
A case of right clavicle mid-shaft fracture after a motorcycle traffic accident, male, 34 years, AO/OTA classification 15.2B. **a** Preoperative radiograph showed right clavicle mid-shaft fracture with wedge fragment, displacement, shortening. **b** Intraoperative photograph, the 2 branches of supraclavicular nerve (yellow arrowhead) were preserved during operation. **c** Postoperative radiograph

All patients underwent standard preoperative evaluation, including X-rays (Fig. 1A), electrocardiography, and laboratory examination. ORIF for clavicle fracture was performed as long as clinical conditions allowed. All operations were performed with the patient under general anesthesia and placed in a semisitting position. Prophylactic antibiotics were administered 30 min prior to incision. The surgical area was sterilized and draped. Before incision, 10–20 mL of anesthetic was administered along the axis of the clavicle, centering the fracture site. Hematoma block was also administered. The anesthetic solution consisted of 20 mL of 2% lidocaine and 1 mL of epinephrine (1:1000) mixed with normal saline to a final concentration of 40 mL (equal to 1% of lidocaine mixed with 1:40,000 epinephrine) [7]. The purpose of the local injection was to decrease oozing during dissection, thereby facilitating in the identification and protection of the supraclavicular nerve.

A direct anterior approach with a longitudinal incision was used in all patients. The incision was either straight or curved, as required and in accordance with each patient’s specific anatomy. The underlying fascia and subcutaneous tissue were split. Hemostasis was performed meticulously. The platysma was incised, and the periosteum was stripped until the fracture site had been adequately exposed. Visible branches of the supraclavicular nerve were identified and protected (Fig. 1B).

After fracture reduction, superior plating with a conventional dynamic compression plate or a locking plate was performed. The reduction and fixation quality were assessed using an image intensifier or postoperative radiograph (Fig. 1C). After copious irrigation, the wound was closed in layers.

The nerve-sacrifice group underwent a similar procedure except that the local injection was not administered and the supraclavicular nerve was not spared.

### Postoperative protocol

The patients used a shoulder sling for 2 weeks postoperatively. An outpatient clinic follow-up was arranged. The sling was discontinued, and unrestricted range-of-motion exercises were allowed. At 6 weeks, the patients were allowed to begin resistance and strengthening exercises if clinical and radiological union was progressing. The patients were asked to avoid contact sports for at least 12 weeks. They were regularly followed up at 4–6-week intervals until complete fracture union. The patients were asked to complete the QuickDASH questionnaire 6 months after their surgery.

### Outcome measurement

The outcome measures included fracture union and time to union, which was detected using follow-up radiographs. Surgical outcomes—including operative time, estimated blood loss, postoperative pain, union rate, time to union, functional score, paresthesia, complications, and implant removal rate—were recorded. Postoperative pain was evaluated using the visual analog scale score on postoperative day 1. Function outcomes were assessed using the QuickDASH scores obtained in the sixth postoperative month during an OPD follow-up; supraclavicular nerve territory numbness was also recorded if present.

Complications after surgery—such as wound infection, nonunion, refracture, and implant-related complications, including backout and implant loosening or breakage—were also recorded.

### Statistics

SPSS (version 26.0; IBM, Armonk, NY, USA) was used for the statistical analysis. Between-group differences were analyzed using independent paired t tests for continuous variables and the Chi-square test for categorical variables. *P* ≤ 0.05 was considered statistically significant.

## Results

**Fig.2:**
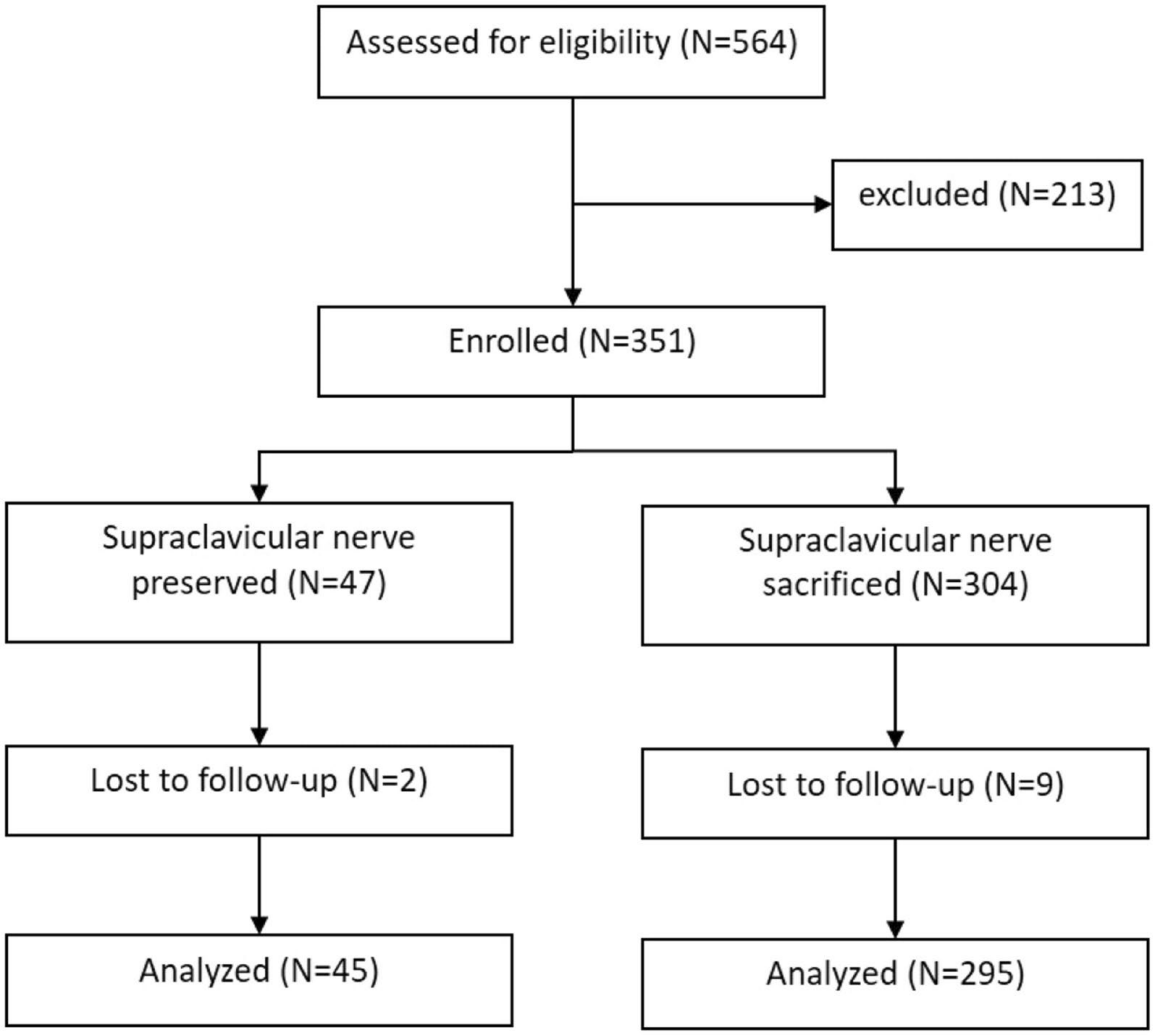
Flow diagram. Excluded (*n* = 213): multiple fractures or polytrauma with other body part injury (*n* = 152), distal clavicle fracture (*n* = 37), proximal clavicle fracture (*n* = 2), chronic fracture (*n* = 6), revision (*n* = 11), under 18 years old 90 (*n* = 4), patient with previous clavicle fracture surgery (*n* = 1)

During the study period, 564 patients underwent ORIF for clavicle fracture at our hospital, 224 of whom were excluded (Fig. 2). The remaining 340 patients were divided into the supraclavicular-nerve-sparing (*n* = 45) and supraclavicular-nerve-sacrifice (*n* = 295) groups. The demographic data are presented in Table 1. The specific outcomes are detailed in Table 2.

**Table 1:** Baseline and demographic characteristics of patients

	Supraclavicular-nerve-preservation group (*N* = 45)	Supraclavicular-nerve-sacrificing group (*N* = 295)	*P* value
Gender			0.633
Male	25 (55.6%)	175 (59.3%)	
Female	20 (44.4%)	120 (40.7%)	
Age, mean ± SD, yr	50.2 ± 16.0 (18–75)	44.6 ± 16.3 (18–82)	0.781
BMI, mean ± SD	24.7 ± 4.2 (17–39.2)	24.6 ± 4.2 (15.6–39.4)	0.925
Injury side			0.106
Right	16 (35.6%)	143 (48.5%)	
Left	29 (64.4%)	152 (51.5%)	
Classification (AO/OTA)			0.548
15.2A	11 (22%)	73 (24.7%)	
15.2B	24 (48%)	135 (45.8%)	
15.2C	10 (20%)	87 (29.5%)	
Trauma mechanism			0.070
Fall	7 (15.6%)	22 (7.5%)	
Motorcycle traffic accident	38 (84.4%)	273 (92.5%)	
Implant			0.488
DCP	9 (18%)	73 (24.7%)	
LCP	36 (72%)	222 (75.3%)	
Days to surgery, mean ± SD, days	3.0 ± 2.5 (1–12)	4.0 ± 3.3 (1–20)	**0.021**
Mean of follow-up time (months)	23.7 ± 7.9 (9–35)	24.2 ± 7.5 (9–35)	0.769

**Table 2:** Outcomes in the preservation group and sacrificing group

	Supraclavicular-nerve-preservation group (*N* = 45)	Supraclavicular-nerve-sacrificing group (*N* = 295)	*P* value
OP time ± SD, min	77.0 ± 18.8 (39–120)	78.4 ± 21.7 (31–138)	0.867
Blood loss ± SD, ml	16.0 ± 13.2 (5–50)	46.6 ± 56.0 (5–300)	** < 0.001**
Union time ± SD, weeks	15.6 ± 4.9 (9–28)	16 ± 5.0 (8–38)	0.614
Pain scale (postop day1)	1.8 ± 1.2 (0–5)	2.1 ± 1.4 (0–8)	0.246
Quick-DASH score 6 m	4.7 ± 5.0 (0–25)	5.2 ± 6.1 (0–43)	0.557
Numbness 6 m			** < 0.001**
(−)	41 (91.1%)	32 (10.8%)	
(+)	4 (8.9%)	263 (89.2%)	
Implant removal rate			0.640
(−)	34 (75.6%)	232 (78.6%)	
(+)	11 (24.4%)	63 (21.4%)	
Revision			
(−)	45 (100%)	295 (100%)	
(+)	0	0	
Complication			0.221
(−)	42 (93.3%)	286 (96.9%)	
(+)	3 (6.7%)	9 (3.1%)	
	Nonunion 1 (2.2%)	Lost of reduction with nonunion 3(1.0%)	
	Superficial infection 1(2.2%)	Superficial infection 2(0.7%)	
	Postoperative stiffness 1(2.2%)	Hardware failure with nonunion 2(0.7%)	
		Post-op pneumothorax 1(0.3%)	
		Periimplant fracture 1(0.3%)	

A total of 340 patients were noted to be eligible. On the basis of operative reports, the patients were divided into supraclavicular-nerve-sparing and supraclavicular-nerve-sacrificing groups. The supraclavicular-nerve-sparing group had 45 patients, and the supraclavicular-nerve-sacrificing group had 295 patients.

The demographic characteristics of the two groups were comparable except for the number of days to surgery, in which the nerve-sparing group averaged 1 day shorter than the nerve-sacrifice group.

The nerve-sparing group had significantly lower rates of incisional or anterior chest wall numbness and less blood loss than the supraclavicular-nerve-sacrifice group. Furthermore, the nerve-sparing group also had nonsignificantly better QuickDASH scores and less postoperative pain than the nerve-sacrifice group. No significant between-group differences were discovered in operative time, union rate, time to union, implant removal rate, complication rate, or revision rate.

## Discussion

Anterior chest wall numbness is the most common complication after ORIF for clavicle fracture [8]. We compared the outcomes between patients undergoing supraclavicular nerve sparing and sacrificing during ORIF of acute midshaft clavicle fracture. Our data indicated that the preservation of supraclavicular nerve branches significantly decreased the likelihood of postoperative incisional and anterior chest wall numbness.

No consensus exists on whether the supraclavicular nerve should be routinely identified and protected during ORIF. Some authors believe that it can be sacrificed when necessary [9]. A 2021 retrospective study demonstrated that although patients may experience some improvement of paresthesia, most experience persistent symptoms [10]. Only a few studies have compared nerve-sparing and nerve-sacrificing techniques. Some studies have reported lower rates of numbness with the nerve-sparing procedure [6, 11].

Vertical incisions have been hypothesized to lead to a lower rate of numbness compared with horizontal incisions, but the results have been inconsistent. One study indicated a lowered rate of numbness [12], but two other studies showed no significant difference between horizontal and vertical incisions [13, 14]. At our institute, we routinely use the horizontal incision due to the extensile exposure and ease of enlargement when necessary.

We also observed that blood loss was significantly less in the nerve-sparing group than in the nerve-sacrifice group. This may have been due to the meticulous dissection during the supraclavicular-nerve dissection and protection as well as the vasoconstriction effect of epinephrine in the WALANT solution. No WALANT-related complications such as cardiac events, seizure, or local skin necrosis were seen. Lidocaine–epinephrine in controlled doses is safe to use [15–17]. Our findings imply that using local injection prior to surgical incision with the WALANT solution is an easy, inexpensive, and effective method of increasing the surgical field and decreasing intraoperative blood loss and postoperative pain.

The nerve-sparing group had nonsignificantly less postoperative pain and slightly better QuickDASH scores, consistent with previous studies [18]. This may have been related to the analgesic effect of the WALANT solution, the meticulous dissection, and the lower likelihood of postoperative numbness. The average QuickDASH score was 4.7 in the nerve-sparing group and 5.2 in the nerve-sacrifice group, which indicates that most patients returned to nearly normal functioning.

We did not observe any between-group differences in surgical time, union rate, union time, complication rate, or implant removal rate. Only one patient had nonunion in the nerve-sparing group, and this nonunion may have been because of suboptimal reduction quality because this was among the first cases for which the nerve-sparing procedure was performed at our institute. The 3 patients who had superficial infection in this study all resolved after surgical debridement and empirical antibiotics treatment uneventfully. The third patient suffered from postoperative stiffness due to secondary frozen shoulder. He regained ROM after prolonged intensive physical therapy. The 5 patients in the sacrificing group with implant-related complications (3 loss of reduction and 2 hardware failure) were all patients who underwent ORIF with conventional DCP. This may be due to the natural disadvantage of the implant design. Pneumothorax was found on an immediate postoperative radiograph in one patient in the sacrificing group. Although no dyspnea or chest pain was complained, a chest tube thoracostomy was performed after consulting a thoracic surgeon. Oxygen supplement was also provided. His pneumothorax resolved uneventfully after serial follow-up. The preoperative radiograph was examined again, but no pneumothorax or rib fractures were found. A 2014 study reported that pneumothorax accounts for 1.2% of complications after clavicle fracture ORIF surgery, but it remains unclear whether pneumothorax is due to injury or surgery [19].

We expected the operative time to be longer in the nerve-sparing group; however, it was comparable in the two groups. This may have been due to the two skillful operators, who now routinely perform supraclavicular nerve sparing during ORIF surgery for acute clavicle fracture. Admittedly, the mean operative time was longer in the first few cases. In our experience, with the help of the WALANT technique and meticulous dissection, nerve sparing can be performed smoothly without jeopardizing reduction and fixation quality after a learning curve of 5–10 cases.

### Surgical tips

Electrocautery will be avoided to decrease the incidence of iatrogenic damage to the nerve. The nerve branches run in the substance of the platysma muscle; care must be taken after entering the muscle during dissection. We will start fracture reduction after ensuring the branches are adequately identified and mobilized.

The fracture reduction and osteosynthesis can only be performed through the ‘windows’ between the nerve branches. Smaller, thinner instruments such as Kelly forceps or pointed towel clips were useful reduction tools. When applying K-wires for provisional fixation or drilling during screw fixation, sleeves must be used to protect the nerve branches.

Operators need to be familiar with the anatomy of the bone, the usual fracture patterns, and the concept of MIPO because sometimes reduction will be obscured by the nerves. Assistants should also be aware that the nerves must be retracted gently.

Implant removal surgery may be considered a revision surgery. We may encounter anatomical changes and adhesions, which will increase the difficulty of preserving the nerve branches. We routinely upload the intraoperative photographs as seen in Fig. 1 to our electronic medical record system. During implant removal surgery, we will make separate small incisions according to the relationship between the screws and nerve branches. If the nerve branches are encountered during dissection, they will be preserved. WALANT solution local injection will also be performed to minimize bleeding and improve the surgical field.

The strength of this study is that it is the largest series conducted on supraclavicular nerve sparing. We did not exclude patients with a complex fracture pattern (AO/OTA 15.2C) or patients with obesity (BMI > 30 kg/m^2^), both of which may increase the difficulty of surgery. Not excluding these patients makes our results more applicable to general practice. We demonstrated that routine supraclavicular nerve sparing in acute clavicle fracture ORIF, regardless of the fracture severity, is feasible. Moreover, the routine use of WALANT solution can improve the surgical field in ORIF of clavicle fracture, decrease intraoperative blood loss, and reduce postoperative pain.

This study also has some limitations. First, the retrospective study design may have resulted in selection bias, data loss, and more confounders than a prospective design would have. Second, the sample size of the nerve-sparing group was relatively small, precluding the identification and analysis of subgroups. Third, only short-term follow-ups have been available so far. Fourth, the two different groups of patients were operated on by two different groups of surgeons. This may alter the surgical results and outcomes. However, all surgeons in our facility were trained in the same system; thus, our surgical techniques were similar. This may decrease some bias. Further prospective studies or randomized controlled trials with larger sample sizes and longer follow-up periods should verify the advantages and necessity of supraclavicular nerve sparing during ORIF.

In conclusion, our study indicated that supraclavicular nerve sparing during ORIF with WALANT can reduce postoperative incisional and anterior chest wall numbness and intraoperative blood loss without increasing the operative time or complication rate.

## Data Availability

The datasets generated and/or analyzed during the current study are available from the corresponding author upon reasonable request.
